# Draft Genome Sequences of Five Staphylococcus saprophyticus Strains Isolated from African Fermented Nono in Nigeria

**DOI:** 10.1128/MRA.00315-21

**Published:** 2021-05-20

**Authors:** Etinosa O. Igbinosa, Erik Brinks, Abeni Beshiru, Isoken H. Igbinosa, Maria Stein, Gyu-Sung Cho, Charles M. A. P. Franz

**Affiliations:** a Applied Microbial Processes and Environmental Health Research Group, Department of Microbiology, Faculty of Life Sciences, University of Benin, Benin City, Nigeria; b Max Rubner Institute, Federal Research Institute of Nutrition and Food, Department of Microbiology and Biotechnology, Kiel, Germany; University of Rochester School of Medicine and Dentistry

## Abstract

Five Staphylococcus saprophyticus strains were isolated from the fermented milk product nono in Nigeria and were sequenced using an Illumina MiSeq platform. The genome sizes ranged from 2.53 to 2.60 Mbp, while the GC contents ranged from 32.99 to 33.07 mol%. The genomes possessed 2,505 to 2,687 protein-coding sequences.

## ANNOUNCEMENT

Coagulase-negative staphylococci (CoNS) are found on the skin and membranes of mammals and birds. These strains are also commonly isolated from different natural niches, such as soil, water, and foods, including meat, cheese, and vegetables (1, 2). Catalase-positive CoNS strains have also been reported to be associated with the raw milk microbiota (3), as have pathogenic coagulase-positive Staphylococcus aureus strains. In this study, the draft genome sequences of five Staphylococcus strains isolated from nono (fermented milk product) samples from the Aduwawa market in Benin City, Nigeria, were determined in order to identify them in an investigation of the microbiological quality and safety of nono. Ten-milliliter nono samples were collected into 90 ml of tryptic soy broth (Oxoid, UK) and incubated at 35°C for 18 to 24 h, and a loopful of culture was streaked onto mannitol salt agar (Neogen Culture Media, Heywood, Lancashire, UK). Five randomly selected colonies were purified by streaking on brain heart infusion (BHI) agar (Roth, Karlsruhe, Germany) at 37°C for 24 h of incubation, and strains (Table 1) were identified as Staphylococcus saprophyticus by 16S rRNA gene sequencing.

**TABLE 1:** *De novo* assembly of strains and draft genome features

Strain^*a*^	No. of contigs	*N*_50_ (bp)	GC content (mol%)	Total length (bp)	Genome coverage (×)	Total no. of paired-end sequence reads	No. of CDSs	No. of tRNAs	No. of rRNAs	Acquired resistance gene(s)	Plasmid database(s)	GenBank accession no.	SRA accession no.
ST386	32	431,815	33.07	2,536,544	65	550,037	2,508	46	5	*cat*(pC233), *dfrg*, *tetK*	Rep_trans, RepA_N	JAFJNU000000000	SRR13674887
ST388	33	218,917	33.00	2,578,047	69	587,237	2,542	48	4	*dfrg*	RepA_N	JAFJNV000000000	SRR13674888
ST390	186	27,155	32.99	2,609,417	85	788,480	2,687	42	8	ND	RepA_N, Inc18	JAFJNW000000000	SRR13674889
ST391	30	431,885	33.07	2,539,289	93	783,645	2,505	45	4	*cat*(pC233), *dfrg*, *tetK*	RepA_N, Rep_trans	JAFJNX000000000	SRR13674890
ST392b	39	373,477	33.04	2,569,600	76	653,274	2,540	47	4	*cat*(pC233), *dfrg*, *tetK*	RepA_N, Rep_trans	JAFJNY000000000	SRR13674891

a All strains were precisely identified as *S. saprophyticus* subsp. *saprophyticus* using the OrthoANI (7) pipeline. CDS, coding sequence; *cat*, chloramphenicol acetyltransferase; *dfrg*, trimethoprim-resistant dihydrofolate reductase; *tet*, tetracycline resistance gene; Rep, replication protein; ND, not detected.

The total genomic DNA of each strain, cultured in BHI broth at 37°C for 18 h, was extracted using a bacterial DNA kit (Peqlab, Erlangen, Germany) according to the manufacturer’s instructions. Genomic DNA libraries were prepared with a Nextera XT library preparation kit (Illumina, USA), and paired-end sequencing was performed on a MiSeq sequencer (Illumina) with 2 × 300 cycles. The total numbers of paired reads ranged from 553,037 to 783,645 (Table 1), and all paired-end raw data were trimmed using the Trimmomatic (v.0.32) (4) pipeline with the following parameters: sliding window; 4:15, leading; 3, and minlen; 45. The *de novo* assemblies were performed with SPAdes (v.3.15.0) with the following parameters: k-mer 77, careful, and minimum contig length of 500 bp (5); the quality of the draft genome sequences obtained was evaluated using the QUAST (v.5.0.2) tool (6). A precise species identification was performed with the OrthoANI (v.0.93.1) (7) pipeline with closely related staphylococcal type strains as references. Acquired antibiotic resistance genes and plasmid-related sequences were identified using ResFinder (v.4.1) (8) and PlasmidFinder (v.2.1) (9), respectively. Except for the SPAdes and Trimmomatic pipelines, default parameters were used for all programs. The *N*_50_ values were between 27,155 and 431,885 bp, and the genome coverage ranged from 65- to 93-fold (Table 1). All contigs were annotated using the PATRIC server and the NCBI Prokaryotic Genome Annotation Pipeline (v.4.13) with default parameters (10–12). The five draft genome sizes range from 2.53 Mbp to 2.60 Mbp, with GC contents between 32.99 and 33.07 mol% (Table 1). Three of the five strains contained genes encoding chloramphenicol (*cat*) and tetracycline (*tetK*) resistance, while replication proteins (Rep) in all five strains indicated the presence of plasmids (Table 1). The details of genome sequencing and annotation results are shown in Table 1.

### Data availability.

The draft genome sequences and the raw read data were deposited under the BioProject accession number PRJNA700496, and the accession numbers are listed in Table 1.
